# Analysis of Parametric and Subharmonic Excitation in Push-Pull Driven Disk Resonator Gyroscopes

**DOI:** 10.3390/mi12010061

**Published:** 2021-01-06

**Authors:** Kai Wu, Kuo Lu, Qingsong Li, Yongmeng Zhang, Ming Zhuo, Sheng Yu, Xuezhong Wu, Dingbang Xiao

**Affiliations:** 1 College of Intelligence Science and Technology, National University of Defense Technology, Changsha 410073, China; wukai@nudt.edu.cn (K.W.); lukuo13@nudt.edu.cn (K.L.); zhangym@nudt.edu.cn (Y.Z.); zhuoming@nudt.edu.cn (M.Z.); fishuup@163.com (S.Y.); xzwu@nudt.edu.cn (X.W.); 2 Hunan MEMS Research Center, Changsha 410073, China; 3 Laboratory of Science and Technology on Integrated Logistics Support, National University of Defense Technology, Changsha 410073, China

**Keywords:** parametric excitation/amplification, quality factor, parametric resonance, subharmonic excitation, push-pull driving method, MEMS disk resonator gyroscope

## Abstract

For micro-electromechanical system (MEMS) resonators, once the devices are fabricated and packaged, their intrinsic quality factors (*Q*) will be fixed and cannot be changed, which seriously limits the further improvement of the resonator’s performance. In this paper, parametric excitation is applied in a push-pull driven disk resonator gyroscope (DRG) to improve its sensitivity by an electrical pump, causing an arbitrary increase of the “effective *Q*”. However, due to the differential characteristics of the push-pull driving method, the traditional parametric excitation method is not applicable. As a result, two novel methods are proposed and experimentally carried out to achieve parametric excitation in the push-pull driven DRGs, resulting in a maximum “effective *Q*” of 2.24 × 10^6^ in the experiment, about a 7.6 times improvement over the intrinsic *Q*. Besides, subharmonic excitation is also theoretically analyzed and experimentally characterized. The stability boundary of parametric excitation, defined by a threshold voltage, is theoretically predicted and verified by related experiments. It is demonstrated that, when keeping the gyroscope’s vibration at a constant amplitude, the fundamental frequency driving voltage will decrease with the increasing of the parametric voltage and will drop to zero at its threshold value. In this case, the gyroscope operates in a generalized parametric resonance condition, which is called subharmonic excitation. The novel parametric and subharmonic excitation theories displayed in this paper are proven to be efficient and tunable dynamical methods with great potential for adjusting the quality factor flexibly, which can be used to further enhance the resonator’s performance.

## 1. Introduction

The disk resonator gyroscope (DRG) is a kind of vibratory gyroscope based on the Coriolis effect, attracting significant attention from MEMS researchers in industry and academia [1]. High precision angular rate measurement and great performance potential make it an admirable inertial gyroscope. For this type of MEMS gyroscopes, its quality factor (*Q*) is one of the most important properties, representing the energy dissipation rate in one oscillation cycle.

There are various factors that lead to the energy dissipation in MEMS gyroscopes, such as the air damping, the surface loss, the thermoelastic damping, and so on [2,3,4]. These damping terms determine the limitation of the gyro’s intrinsic *Q* and have already been fixed during the processing. In this case, we consider pumping energy into the vibration modes to enhance their “effective *Q*” and hence improve the device’s sensitivity [5,6,7,8].

Moreover, the nonlinearity of the MEMS gyroscopes becomes significant due to the continuous size reduction, which has been deeply investigated in the recent past [9,10,11,12,13]. There are not only negative effects that must be suppressed, but also useful properties that can be exploited in nonlinear MEMS resonators [10]. Most of the MEMS resonators are operated in the linear regime to avoid hysteresis and additional noise associated with nonlinearities, in which the double hysteresis behavior is caused by the electrostatic and mechanical nonlinearities [11]. Besides, electrostatic nonlinear mode coupling is very common in capacitive MEMS resonators [14]. Research in a high-order nonlinear MEMS resonator demonstrates that the parametric noise can be suppressed and the frequency stability can be improved when operated at two of its bifurcation points [11]. The dynamic characteristics and bifurcation analysis were investigated in a 4-DOF micro gyroscope [12], and the influence of nonlinearity on the phase characteristics was analyzed [13].

Parametric excitation is a technique to enhance the “effective *Q*” by pumping energy into the oscillation system [15], which is usually realized by modulating the device’s stiffness with the double resonance frequency signals [16]. In this condition, the gyroscope can be modeled as a driven damped harmonic oscillator with a time-dependent dynamic stiffness, whose motion can be described by the Mathieu–Hill equation [17], as shown in Equation (1).

(1) $$m\ddot{q}+\frac{m\omega_{n}}{Q}\dot{q}+\left[k_{0}+\Delta k\sin(2\omega_{d}t+\phi )\right]q=F(t)$$

Here, *ω_n_* is the natural frequency, *ω_d_* is the driving frequency, *k*_0_ is the initial stiffness, and Δ*k* is the stiffness modulation. Experiments in previous studies have proven that the “effective *Q*” of the gyroscope can be tuned by parametric excitation signals [16,18,19,20,21,22,23,24,25], indicating that the parametric pump can not only enhance the “effective *Q* factor”, but also suppress it. Because the effect of parametric excitation is phase-sensitive, the parametric pump will amplify the oscillation at a particular phase, but squeeze it at the inverter [26,27].

Generally, it is difficult to detect the signal of angular velocity due to the small resonant mass and weak Coriolis force, which lead to the decrease of the gyro’s sensitivity. Parametric excitation in sense mode provides an approach to amplify the Coriolis response directly, making an improvement in the scale factor. Besides, this amplification occurs before the addition of the noise of the first electronic stage, which is a particular advantage for noise squeezing, cause that electronic noise is the major noise contributor for these kinds of MEMS sensors [28]. In this case, the output of the gyro is increased, while the circuit noise maintains the same level, which indicates the particular advantage of the improvement of the signal-to-noise ratio (SNR). The open-loop parametric amplification of sense mode was demonstrated in an encapsulated DRG, resulting in an 8.8 times scale factor improvement [18]; and the SNR improved by a factor of 9.5 [20].

In push-pull circuits, the drive signal is usually applied to one pair of electrodes, and its reverse signal is simultaneously applied to the other pair of differential electrodes. Under these circumstances, although the excitation efficiency can be sufficiently improved, the parametric amplification becomes invalid when the parametric pumps are applied to the same differential electrodes in the same way. This is because the working terms for parametric amplification are eliminated due to the differential characteristics of the push-pull circuits. Therefore, although these ring gyroscopes have multiple symmetrically distributed electrodes, the parametric excitation pump was coincidentally applied on a single electrode in previous studies [16,18,19,20]. In this case, the amplification efficiency is limited and unsatisfactory.

Moreover, it has been noted that there is a stability boundary in the parametric excitation process, which determines the stability and the maximum efficiency of parametric amplification [16,19,20]. When the parametric excitation voltage *V_p_* is larger than the threshold *V_th_*, sustained oscillations, called the parametric resonance, will occur [5,16]. Specifically, when the driving force of the fundamental frequency withdraws while the double frequency parametric excitation signal remains at the threshold level (or even larger), the gyroscope will keep vibrating. In this case, the gyroscope operates in a generalized parametric resonance condition, which is called subharmonic excitation.

In this paper, parametric excitation and subharmonic excitation in the disk resonator gyroscopes are theoretically analyzed by the experiment results. Firstly, the structure and dynamic model of the DRG are introduced in Section 2. Then, the analysis of the basic laws of parametric excitation in push-pull driven circuits is carried out in Section 3. In this part, to take advantage of the push-pull driving method and parametric amplification, two modified methods are presented. The threshold voltage for the stability boundary of parametric excitation and subharmonic excitation is theoretically analyzed in Section 4. Furthermore, Section 5 presents the related experimental results of the DRG’s parametric amplification and subharmonic excitation. Finally, the basic principles for parametric excitation and subharmonic excitation in disk resonator gyroscopes are concluded in Section 6.

## 2. The Device Description and Dynamic Model of the DRG

**Figure 1:**
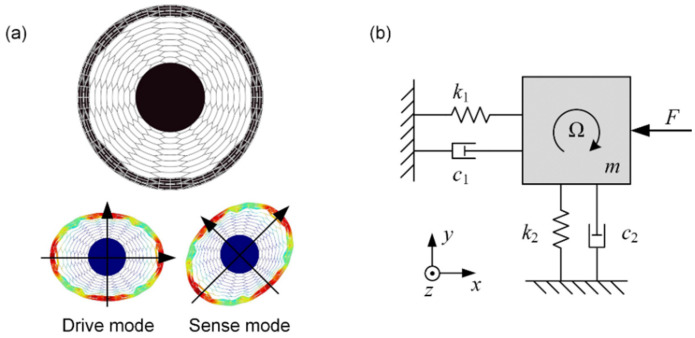
(**a**) The resonant structure of the honeycomb-like disk resonator gyroscope (DRG) and its working modes; (**b**) the two DOF equivalent model of the DRG.

The disk resonator gyroscope is one kind of typical axisymmetric gyroscope operating in two elliptical modes, which are drive mode and sense mode, respectively. In this paper, a honeycomb-like disk resonator gyroscope was chosen for this study, and it works in the *n* = 2 elliptical mode as shown in Figure 1a. This kind of DRG inherits many advantages from the traditional DRG and owns unique characteristics due to its topology structure, such as higher immunity to fabrication errors, better resonant mode consistency, and better inner electrode arrangement [29]. Ideally, these two modes have an identical natural frequency with the same mode shapes, which is convenient to achieve mode matching [1]. When the external angular velocity acts on the gyroscope, the sense mode of the gyroscope in the orthogonal direction will be excited and sense the changing of the angular velocity. The gyroscope can be equivalently modeled as a two-degree-of-freedom system, which can be represented by a spring-mass-damper in each orthogonal direction [30] as shown in Figure 1b. The simplified dynamic equation in the ideal state can be described as:

(2) $$\begin{array}{l} m\ddot{x}+c_{1}\dot{x}+k_{1}x=F(t)\\ m\ddot{y}+c_{2}\dot{y}+k_{2}y=-2nA_{g}m\Omega \dot{x} \end{array}$$

where *m*, *c*, and *k* are the effective vibration mass, damping coefficient, and stiffness of each mode, respectively; *A_g_* is the angular gain, and for *n* = 2 degenerate mode, *A_g_* ≈ 0.37; and Ω is the outside angular rate.

Generally, for the ideal model of the DRG (*c*_1_ = *c*_2_ and *k*_1_ = *k*_2_), the mechanical sensitivity can be obtained by solving Equation (2):

(3) $$S_{mech}=\frac{y}{\Omega }=\frac{4A_{g}Q}{\omega_{n}}\left|x\right|=2A_{g}\tau \left|x\right|$$

where |x| is the oscillation amplitude of drive mode, and *τ* is the decaying time constant that satisfies *τ* = 2*Q*/*ω_n_*.

## 3. Parametric Excitation in the Push-Pull Driven DRG

### 3.1. Parametric Excitation by a Single Electrode

Compared with the traditional resonant excitation methods where generally only fundamental frequency excitation signals are applied to the system, fundamental frequency driving signals are applied to the gyroscope associated with pump signals simultaneously. The frequency of the pump signal *ω_p_* satisfies the condition of *ω_p_* ≈ 2*ω_n_*/*l*, where *ω_n_* is the natural frequency of the system, and *l* is a positive integer [16,17]. The first order of *l* = 1 is considered in this paper. In the case of parametric excitation, the gyroscope’s response can be amplified by a small parametric excitation pump, which appears as a dynamical parameter in the gyroscope’s governing equation [11].

**Figure 2:**
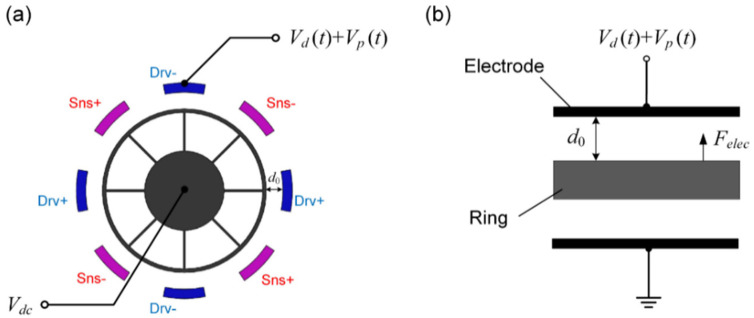
The parametric excitation signals applied to a single electrode. (**a**) The simplified ring structure and the diagram for parametric excitation; (**b**) the equivalent dynamic model of the DRG when actuated by a single electrode.

In the previous study where parametric excitation was applied to a ring gyroscope, parametric excitation signals were commonly applied to a single electrode, as shown in Figure 2. In this case, the electrostatic driving force can be calculated as Equation (4) after the Taylor expansion:

(4) $$\begin{array}{ll} F_{elec} & =\frac{\varepsilon_{r}\varepsilon_{0}A_{eff}}{2{\left(d_{0}-x\right)}^{2}}{\left[V_{dc}+V_{d}\sin\omega_{d}t+V_{p}\sin\left(\omega_{p}t+\phi \right)\right]}^{2}\\ & =\sum_{j=0}^{\infty }K_{j}(t)x^{j} \end{array}$$

where:

(5) $$K_{j}(t)=\varepsilon_{r}\varepsilon_{0}A_{eff}\frac{j+1}{2d_{0}^{j+2}}{\left[V_{dc}+V_{d}\sin\omega_{d}t+V_{p}\sin\left(\omega_{p}t+\phi \right)\right]}^{2}$$

where *ε_r_* is the relative permittivity, *ε*_0_ is the vacuum dielectric constant, and *d*_0_ is the initial capacitive clearance. In this case, a DC voltage bias *V_dc_* is applied to the device through the center anchor, and a sinusoidal voltage *V_d_*(*t*) = *V_d_*sin*ω_d_t* superposed with a pump signal *V_p_*(*t*) = *V_p_*sin(*ω_p_t* + *ϕ*) is applied to the drive electrode, where *ω_p_* = 2*ω_d_*. *A_eff_* is the equivalent capacitance area between the drive electrodes and the resonant structure.

Generally, the displacement of the ring is limited to much smaller than the gap to avoid the pull-in effect. Moreover, the terms of higher orders (*j* ≥ 2) in Equation (4) can be neglected in this condition, and the expression of the electrostatic driving force can be written as:

(6) $$F_{elec}\approx F_{0}(t)+K(t)x$$

where:

(7) $$\begin{array}{l} F_{0}(t)=\frac{\varepsilon_{r}\varepsilon_{0}A_{eff}}{d_{0}^{2}}V_{dc}V_{d}\sin\omega_{d}t\\ K(t)=\frac{\varepsilon_{r}\varepsilon_{0}A_{eff}}{2d_{0}^{3}}\left[(2V_{dc}^{2}+V_{d}^{2}+V_{p}^{2})+4V_{dc}V_{p}\sin(2\omega_{d}t+\phi )+2V_{d}V_{p}\sin\omega_{d}t\sin(2\omega_{d}t+\phi )-V_{d}^{2}\cos2\omega_{d}t\right] \end{array}$$

Considering that generally 4*V_dc_V_p_* > 10,000$V_{d}^{2}$ in real experiment settings and the component of sin*ω_d_t*sin(*ω_p_t* + *ϕ*) does not work for parametric excitation either [11], the expression of *K*(*t*) can be further simplified as:

(8) $$K(t)\approx \frac{\varepsilon_{r}\varepsilon_{0}A_{eff}}{2d_{0}^{2}}\left[\left(2V_{dc}^{2}+V_{d}^{2}+V_{p}^{2})+4V_{dc}V_{p}\sin(2\omega_{d}t+\phi \right)\right]$$

Substituting (6)–(8) into the dynamic Equation (2) of the DRG, we can obtain the Mathieu–Hill equation:

(9) $$\ddot{x}+\frac{\omega_{1}}{Q_{1}}\dot{x}+\left[\omega_{1}^{2}-H_{1}-H_{2}\sin(2\omega_{d}t+\phi )\right]x=H_{3}\sin\omega_{d}t$$

where:

(10) $$\begin{array}{l} \beta_{1}=\frac{\varepsilon_{r}\varepsilon_{0}A_{eff}}{2md_{0}^{3}},\beta_{2}=\frac{\varepsilon_{r}\varepsilon_{0}A_{eff}}{md_{0}^{2}}\\ H_{1}=(2V_{dc}^{2}+V_{d}^{2}+V_{p}^{2})\beta_{1}\\ H_{2}=4V_{dc}V_{p}\beta_{1}\\ H_{3}=V_{dc}V_{d}\beta_{2} \end{array}$$

Here, *ω*_1_ is the natural frequency and *Q*_1_ is the quality factor of the drive mode, respectively. Moreover, we noticed that *H*_1_ is a constant term related to the square of the DC voltages, and it appears as a coefficient of *x* in the dynamic equation, which is directly related to the stiffness of the system. In this case, the natural frequency of the resonator will go down due to the applied voltages. Based on the harmonic balance method [17,31], considering that the motion of drive mode is approximately periodic, the steady-state solution can be written as a Fourier series:

(11) $$x(t)=\sum_{k=1}^{\infty }\left(a_{k}\cos k\omega_{d}t+b_{k}\sin k\omega_{d}t\right)$$

Substituting (11) into (9) and equating the coefficients of cos*kωt* and sin*kωt* on the two sides of “=” respectively, two sets of inhomogeneous equations can be obtained:

(12) $$\begin{array}{l} A_{1}a+B_{1}b=c_{1}\\ A_{2}a+B_{2}b=c_{2} \end{array}$$

where:

(13) $$\begin{array}{l} A_{1}=\left[\begin{array}{ccccc} -\zeta_{1}\omega_{d} & 0 & \frac{-H_{2}\cos\phi }{2} & & \\ 0 & -2\zeta_{1}\omega_{d} & 0 & \ddots & \\ \frac{H_{2}\cos\phi }{2} & 0 & -3\zeta_{1}\omega_{d} & \ddots & \frac{-H_{2}\cos\phi }{2}\\ & \ddots & \ddots & \ddots & 0\\ & & \frac{H_{2}\cos\phi }{2} & 0 & -k\zeta_{1}\omega_{d} \end{array}\right]\\ B_{1}=\left[\begin{array}{ccccc} {\bar{\omega }}_{1}^{2}-\omega_{d}^{2} & 0 & \frac{H_{2}\sin\phi }{2} & & \\ 0 & {\bar{\omega }}_{1}^{2}-4\omega_{d}^{2} & 0 & \ddots & \\ \frac{H_{2}\sin\phi }{2} & 0 & {\bar{\omega }}_{1}^{2}-9\omega_{d}^{2} & \ddots & \frac{H_{2}\sin\phi }{2}\\ & \ddots & \ddots & \ddots & 0\\ & & \frac{H_{2}\sin\phi }{2} & 0 & {\bar{\omega }}_{1}^{2}-{\left(k\omega_{d}\right)}^{2} \end{array}\right]\\ A_{2}=\left[\begin{array}{ccccc} {\bar{\omega }}_{1}^{2}-\omega_{d}^{2} & 0 & \frac{H_{2}\sin\phi }{2} & & \\ 0 & {\bar{\omega }}_{1}^{2}-4\omega_{d}^{2} & 0 & \ddots & \\ \frac{H_{2}\sin\phi }{2} & 0 & {\bar{\omega }}_{1}^{2}-9\omega_{d}^{2} & \ddots & \frac{H_{2}\sin\phi }{2}\\ & \ddots & \ddots & \ddots & 0\\ & & \frac{H_{2}\sin\phi }{2} & 0 & {\bar{\omega }}_{1}^{2}-{\left(k\omega_{d}\right)}^{2} \end{array}\right]\\ B_{2}=\left[\begin{array}{ccccc} \zeta_{1}\omega_{d} & 0 & \frac{H_{2}\cos\phi }{2} & & \\ 0 & 2\zeta_{1}\omega_{d} & 0 & \ddots & \\ \frac{-H_{2}\cos\phi }{2} & 0 & 3\zeta_{1}\omega_{d} & \ddots & \frac{H_{2}\cos\phi }{2}\\ & \ddots & \ddots & \ddots & 0\\ & & \frac{-H_{2}\cos\phi }{2} & 0 & k\zeta_{1}\omega_{d} \end{array}\right]\\ a={\left[\begin{array}{cccc} a_{1} & a_{2} & \cdots & a_{k} \end{array}\right]}^{T}\\ b={\left[\begin{array}{cccc} b_{1} & b_{2} & \cdots & b_{k} \end{array}\right]}^{T}\\ c_{1}={\left[\begin{array}{ccccc} H_{3} & 0 & 0 & \cdots & 0 \end{array}\right]}^{T}\\ c_{2}={\left[\begin{array}{cccc} 0 & \cdots & 0 & 0 \end{array}\right]}^{T}\\ \zeta_{1}=\frac{\omega_{1}\omega_{d}}{Q_{1}}\\ {\bar{\omega }}_{1}^{2}=\omega_{1}^{2}-H_{1}\\ {\hat{\omega }}_{1}^{2}={\bar{\omega }}_{1}^{2}-\omega_{d}^{2} \end{array}$$

Here, ${\bar{\omega }}_{1}$ represents the modulated frequency of the DRG, which has been decreased by the applied voltages, and ${\hat{\omega }}_{1}$ represents the mistuning between the driving frequency *ω_d_* and the DRG’s modulated frequency ${\bar{\omega }}_{1}$ [16]. Neglecting high-order harmonic signals, the coefficients of (11) can be calculated as:

(14) $$a_{1}=\frac{-4\zeta_{1}+2H_{2}\cos\phi }{4{\hat{\omega }}_{1}^{4}+4\zeta_{1}^{2}-H_{2}^{2}},b_{1}=\frac{4{\hat{\omega }}_{1}^{2}-2H_{2}\sin\phi }{4{\hat{\omega }}_{1}^{4}+4\zeta_{1}^{2}-H_{2}^{2}}$$

As a result, the steady-state solution of Equation (9) can be expressed as:

(15) $$x(t)=A_{1}\sin(\omega_{d}t+\psi_{1})$$

where:

(16) $$\begin{array}{l} A_{1}=\frac{2H_{3}\sqrt{4{\hat{\omega }}_{1}^{4}+4\zeta_{1}^{2}+H_{2}^{2}-4H_{2}({\hat{\omega }}_{1}^{2}\sin\phi +\zeta_{1}\cos\phi )}}{4{\hat{\omega }}_{1}^{4}+4\zeta_{1}^{2}-H_{2}^{2}}\\ \psi_{1}=\tan^{-1}\left(-\frac{2\zeta_{1}-H_{2}\cos\phi }{2{\hat{\omega }}_{1}^{2}-H_{2}\sin\phi }\right) \end{array}$$

According to Equation (16), the parametrically amplified amplitude *A*_1_ is a function of the frequency mistuning ${\hat{\omega }}_{1}$, the parametric voltage *V_p_*, and the phase advance *ϕ*. The maximum amplitude of *A*_1_ appears at *ϕ* = ±*π*, while the amplitude will be suppressed when *ϕ* = 0, indicating that parametric excitation is phase-sensitive. As a result, the “effective *Q*” can be tuned by modifying the phase *ϕ*.

It is also apparent from Equation (16) that the following condition has to be satisfied for a stable oscillation of the DRG when parametrically excited:

(17) $$4{\hat{\omega }}_{1}^{4}+4\zeta_{1}^{2}-H_{2}^{2}>0$$

When it approaches zero, the oscillation amplitude will increase without bound in open-loop driving mode. What is more, the stability boundary of the system is also determined by Equation (16), and it will be discussed in detail in Section 4.

To evaluate the magnification of the parametric amplification, the parametric amplification gain factor *G*_1_ is defined as:

(18) $$G_{1}=\frac{A_{1}{|}_{V_{p}\neq 0}}{A_{1}{|}_{V_{p}=0}}=\frac{2\zeta_{1}}{2\zeta_{1}-H_{2}}$$

It represents the ratio of the amplitude with and without parametric excitation under the condition of the maximum oscillation amplitude (${\hat{\omega }}_{1}^{2}=0$ and *ϕ* = ±*π*), which is a function of parametric voltage *V_p_*. Substituting (10) and (13) into (18), it can be obtained that there is a linear relationship between the reciprocal of *G*_1_ and the parametric excitation voltage *V_p_*:

(19) $$\frac{1}{G_{1}}=1-\frac{H_{2}}{2\zeta_{1}}=1-\left(\frac{2\beta_{1}V_{dc}Q_{1}}{\omega_{1}\omega_{d}}\right)V_{p}$$

### 3.2. Parametric Excitation in Traditional Push-Pull Driving

**Figure 3:**
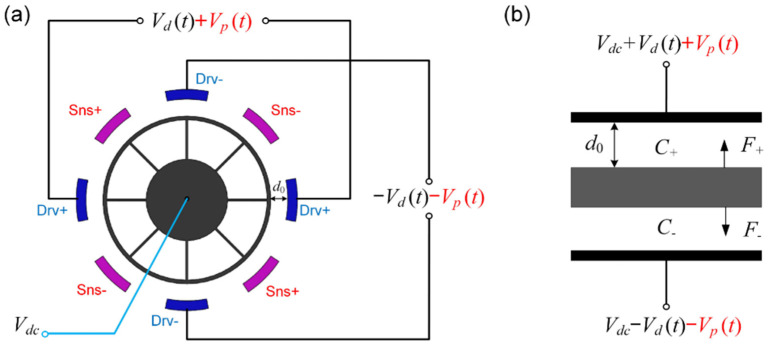
The parametric excitation signals applied to the differential electrodes. (**a**) The simplified ring structure and the diagram for parametric excitation; (**b**) the equivalent dynamic model of the DRG when actuated by the push-pull driving force.

To reduce the asymmetry errors and to improve the driving efficiency, the excitation signals are typically applied to two pairs of differential electrodes based on the push-pull driving method, as shown in Figure 3. The parametric excitation signals and driving signals are superimposed together, and then, their in-phase signals and inverted signals are simultaneously applied to the differential electrodes. In this case, the effective capacitive area doubles, and the electrostatic driving force is calculated as (20), where $A_{\mathrm{eff}}^{\prime }=2A_{\mathrm{eff}}$.

(20) $$F_{elec}^{\prime }=\frac{\varepsilon_{r}\varepsilon_{0}A_{eff}^{\prime }}{2{\left(d_{0}-x\right)}^{2}}{\left[V_{dc}+V_{d}\sin\omega_{d}t+V_{p}\sin\left(\omega_{p}t+\phi \right)\right]}^{2}-\frac{\varepsilon_{r}\varepsilon_{0}A_{eff}^{\prime }}{2{\left(d_{0}+x\right)}^{2}}{\left[V_{dc}-V_{d}\sin\omega_{d}t-V_{p}\sin\left(\omega_{p}t+\phi \right)\right]}^{2}$$

Similarly, after the Taylor expansion and neglecting the high-order terms, the driving force can be written as:

(21) $$F_{elec}^{\prime }\approx F_{0}^{\prime }(t)+K^{\prime }(t)x$$

where:

(22) $$\begin{array}{l} F_{0}^{\prime }(t)=\frac{\varepsilon_{r}\varepsilon_{0}A_{eff}^{\prime }}{d_{0}^{2}}2V_{dc}V_{d}\sin\omega_{d}t\\ K^{\prime }(t)=\frac{\varepsilon_{r}\varepsilon_{0}A_{eff}^{\prime }}{d_{0}^{3}}\left[\left(2V_{dc}^{2}+V_{d}^{2}+V_{p}^{2})+4V_{d}V_{p}\sin\omega_{d}t\sin(2\omega_{d}t+\phi \right)\right] \end{array}$$

It is obvious that there are no terms that can work for parametric excitation in the expression of the driving force, as the effective terms of double frequency are symmetrically eliminated during the differential excitation process. In other words, parametric excitation has failed in traditional push-pull driven gyroscopes. Therefore, to combine the advantages of the push-pull driving method and parametric excitation, two novel methods are proposed to achieve parametric excitation in push-pull driven gyroscopes.

#### 3.2.1. Triple-Frequency Parametric Excitation in Push-Pull Driving

The first method is the triple-frequency parametric excitation method, where the frequency of the parametric excitation signal is three times the resonant frequency instead of the traditional double one. The schematic diagram of this method is consistent with parametric excitation in the traditional push-pull driving method, as shown in Figure 3, only with the changing of the pump frequency from double frequency to triple frequency. Under these circumstances, *ω_p_* = 3*ω_d_*, and only in this frequency can a term of double frequency be generated in *K*′(*t*) as shown in Equation (23), which can be used to achieve parametric excitation.

(23) $$\begin{array}{ll} K^{\prime }(t) & =\frac{\varepsilon_{r}\varepsilon_{0}A_{eff}^{\prime }}{d_{0}^{3}}\left[\left(2V_{dc}^{2}+V_{d}^{2}+V_{p}^{2})+4V_{d}V_{p}\sin\omega_{d}t\sin(3\omega_{d}t+\phi \right)\right]\\ & =\frac{\varepsilon_{r}\varepsilon_{0}A_{eff}^{\prime }}{d_{0}^{3}}\left[\left(2V_{dc}^{2}+V_{d}^{2}+V_{p}^{2})-2V_{d}V_{p}\cos(4\omega_{d}t+\phi )+2V_{d}V_{p}\cos(2\omega_{d}t+\phi \right)\right] \end{array}$$

Neglecting the term of 4*ω_d_* that does not work for parametric excitation and letting *Φ* = *ϕ* + *π*/*2*, Equation (23) can be described by:

(24) $$K^{\prime }(t)=\frac{\varepsilon_{r}\varepsilon_{0}A_{eff}^{\prime }}{d_{0}^{3}}\left[\left(2V_{dc}^{2}+V_{d}^{2}+V_{p}^{2})+2V_{d}V_{p}\sin(2\omega_{d}t+\Phi \right)\right]$$

In this case, the reciprocal of the parametric gain is calculated as:

(25) $$\frac{1}{G_{1}^{\prime }}=1-\left(\frac{4\beta_{1}Q_{1}}{\omega_{1}\omega_{d}}\right)V_{d}V_{p}$$

In this case, the term sin*ω_d_t*sin(*ω_p_t* + *ϕ*) will produce a double frequency component when *ω_p_* = 3*ω_d_*, while this term did not operate previously in the traditional parametric excitation condition where *ω_p_* = 2*ω_d_*. Specifically, this term will produce the effective stiffness modulation term of parametric excitation only when *ω_p_* = 3*ω_d_*, indicating that other frequencies such as 2, 4, and 5 times have no parametric amplification effect. With this novel method, parametric excitation and the push-pull driven method can be applied at the same time without changing the circuit driving system, effectively improving the driven efficiency and sensitivity.

Comparing Equation (19), as for this method, it is obvious that the efficiency of the amplification depends on the magnitude of the driving voltage *V_d_*, while it is generally much smaller than the DC bias *V_dc_*. Although the efficiency of the amplification is smaller than that in the single electrode driven method, this method is the first to successfully combine parametric excitation with the push-pull driven method, which realizes the joint improvement of the driving efficiency and amplification gain. This method provides a new way to achieve the parametric amplification by using a triple frequency pump signal in the push-pull driven method, which reveals a significant potential to reduce the parasitic signals in capacitive sensing and improve the gyroscope’s sensitivity.

#### 3.2.2. The Non-Differential Parametric Excitation in Push-Pull Driving

**Figure 4:**
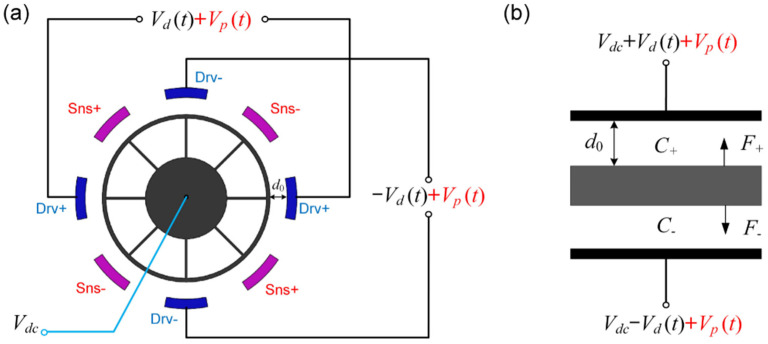
The non-differential parametric excitation signals applied to the differential electrodes. (**a**) The simplified ring structure and the diagram for parametric excitation; (**b**) the equivalent dynamic model of the DRG when actuated by the push-pull driving force.

The second method is the non-differential parametric excitation that applies the same double frequency signals to the driving electrodes without the differential transformation, as shown in Figure 4. As a result, the in-phase parametric excitation signals are applied on the differential electrodes, which ensures that the effective parametric amplification signals are not offset.

In this case, the mixed excitation force can be described by:

(26) $$\begin{array}{ll} F_{elec}^{\prime \prime } & =\frac{\varepsilon_{r}\varepsilon_{0}A_{eff}^{\prime }}{2{\left(d_{0}-x\right)}^{2}}{\left[V_{dc}+V_{d}\sin\omega_{d}t+V_{p}\sin\left(\omega_{p}t+\phi \right)\right]}^{2}\\ & -\frac{\varepsilon_{r}\varepsilon_{0}A_{eff}^{\prime }}{2{\left(d_{0}+x\right)}^{2}}{\left[V_{dc}-V_{d}\sin\omega_{d}t+V_{p}\sin\left(\omega_{p}t+\phi \right)\right]}^{2}\\ & \approx F_{0}^{\prime \prime }(t)+K^{\prime \prime }(t)x \end{array}$$

where:

(27) $$\begin{array}{l} F_{0}^{\prime \prime }(t)=\frac{\varepsilon_{r}\varepsilon_{0}A_{eff}^{\prime }}{d_{0}^{2}}\left[2V_{dc}V_{d}\sin\omega_{d}t+V_{d}V_{p}\cos(\omega_{d}t+\phi )\right]\\ K^{\prime \prime }(t)=\frac{\varepsilon_{r}\varepsilon_{0}A_{eff}^{\prime }}{d_{0}^{3}}\left[\left(2V_{dc}^{2}+V_{d}^{2}+V_{p}^{2})+4V_{dc}V_{p}\sin(2\omega_{d}t+\phi \right)\right] \end{array}$$

In this case, the effect of parametric amplification depends on the DC bias voltage *V_dc_*, which is larger than the driving voltage *V_d_*. Therefore, only a smaller parametric excitation voltage is required to reach the same effect of amplification compared with the triple frequency parametric excitation. The efficiency of the amplification improved sufficiently. Considering that generally 2*V_dc_* > 100*V_p_*, the term *V_d_V_p_*cos(*ω_d_t* + *ϕ*) can be neglected, and the gain factor of non-differential parametric amplification is expressed as:

(28) $$\frac{1}{G_{1}^{\prime \prime }}=1-\left(\frac{8\beta_{1}V_{dc}Q_{1}}{\omega_{1}\omega_{d}}\right)V_{p}$$

Compared with the traditional parametric excitation by a single electrode, the parametric gain factor of the non-differential excitation in the push-pull driven method improved by:

(29) $$K=\frac{G_{1}^{\prime \prime }}{G_{1}}=1+\frac{6\beta_{1}}{\frac{\zeta_{1}}{V_{dc}V_{p}}-8\beta_{1}}$$

As mentioned above, *β*_1_ is a coefficient related to the DRG’s geometric parameters, and *ζ*_1_ is determined by the natural frequency and quality factor of the DRG. As shown in Equation (29), the specific value of *K*, representing the enhancement of non-differential parametric excitation, has a more significant effect when the parametric voltage *V_p_* is held at a higher level.

## 4. Subharmonic Resonance

### 4.1. The Analysis of the Threshold Voltage in Parametric Excitation

According to Equation (16), the sustained oscillation occurs when the vibration system satisfies:

(30) $$4{\hat{\omega }}_{1}^{4}+4\zeta_{1}^{2}-H_{2}^{2}=0$$

Theoretically, when the above-mentioned equation is satisfied, the amplitude of the gyroscope will become infinite, and it will no longer be a steady-state response. In this case, the gyroscope operates in a parametric resonance condition, which determines the stability boundary of the parametrically excited systems [16]. The threshold between the steady-state and the unsteady-state can be obtained by solving Equation (32):

(31) $$V_{t}=\frac{\omega_{1}\omega_{d}}{2\beta_{1}Q_{1}V_{dc}}$$

When *V_p_* = *V_t_*, the gyroscope will sustain its vibrating even if the driving signal *V_d_*(*t*) is removed. Moreover, the oscillation amplitude will increase without bound theoretically when *V_p_* > *V_t_*, but it will be limited by the pull-in effect when its amplitude reaches a certain level in the real system. It is demonstrated that the “effective” *Q*-factor will grow infinitely with the input of parametric energy.

### 4.2. The Analysis of Subharmonic Excitation

When the fundamental driving signals are removed while the double frequency parametric excitation signals remain at the threshold voltage level (or even larger), the gyroscope will enter a special parametric resonance condition called subharmonic excitation. In general, the appearance of subharmonic excitation is caused by the gyroscope’s nonlinearities, and the frequency multiplier of the harmonic excitation is consistent with the order of the functioning nonlinearity. In this paper, the DRG’s subharmonic resonance caused by the quadratic nonlinearity is analyzed.

Previous research demonstrated that electrostatic nonlinearity is the main nonlinearity in disk MEMS gyroscopes, which is caused by electrostatic forces [32]. The nonlinear dynamic model of the gyroscope is usually represented as:

(32) $$m\ddot{x}+c\dot{x}+(k_{0}+k_{1})x+k_{2}x^{2}+k_{3}x^{3}=F_{0}\cos(\Omega t)$$

Here, *k*_0_ is the inherent mechanical elastic coefficient, while *k*_1_, *k*_2_, and *k*_3_ are the gyroscope’s first-order, second-order, and third-order nonlinear elastic coefficient, respectively, which can be expressed as:

(33) $$\{\begin{array}{ll} k_{1}=\frac{2\varepsilon_{0}\varepsilon_{r}AV_{dc}^{2}}{d_{0}^{3}}, & k_{2}=\frac{3\varepsilon_{0}\varepsilon_{r}AV_{dc}^{2}}{d_{0}^{4}}\\ k_{3}=\frac{4\varepsilon_{0}\varepsilon_{r}AV_{dc}^{2}}{d_{0}^{5}}, & F_{0}=\frac{2\varepsilon_{0}\varepsilon_{r}AV_{dc}V_{d}}{d_{0}^{2}} \end{array}$$

Traditional methods are not suitable for solving high-order nonlinear equations for which the multi-scale method is applied to analyze this oscillation system. Firstly, we unify the dimensions of the items in Equation (32) by introducing a small parameter *ε*. Then, we extend the traditional time scale of the system to multiple time scales, where *T*_0_ = *t*, *T*_1_ = *εt*. Therefore, the differential Equation (32) can be rewritten as:

(34) $$\ddot{u}+\omega_{0}^{2}u=-2\varepsilon \mu \dot{u}-\varepsilon \alpha_{2}u^{2}-\varepsilon^{2}\alpha_{3}u^{3}+f_{0}\cos\Omega t$$

When the excitation signal’s frequency satisfies Ω = 2*ω*_0_ + *εσ*, where *σ* is the frequency detuning, based on the perturbation method, the solution of Equation (34) can be calculated as:

(35) $$u=a\exp(\lambda \varepsilon t)\cos\left(\frac{1}{2}\Omega t\pm \theta \right)+\frac{f_{0}}{\omega_{0}^{2}-\Omega^{2}}\cos(\Omega t)$$

where:

(36) $$\lambda =-\mu \pm \sqrt{\frac{\alpha_{2}^{2}f_{0}^{2}}{4\omega_{0}^{2}{\left(\omega_{0}^{2}-\Omega^{2}\right)}^{2}}-\frac{\sigma^{2}}{4}}$$

**Figure 5:**
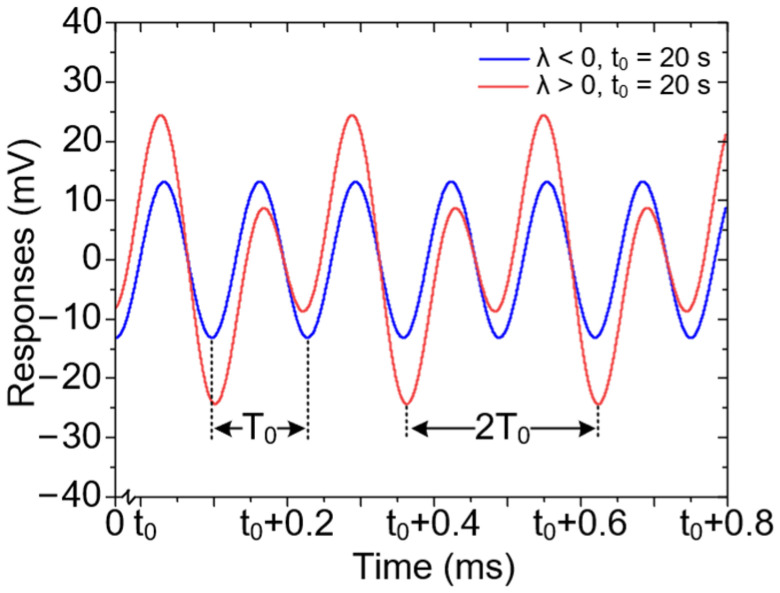
Simulation results of the disk MEMS gyroscope’s subharmonic responses when it has quadratic nonlinearity.

It is clear that the gyroscope’s response contains a steady-state term (double frequency response) and a time-varying term (fundamental frequency response), whose evolutionary principle is related to the value of *λ*. When *λ* is a complex number, the amplitude of the fundamental frequency response is a continuous oscillation attenuation term; when *λ* is a real number and *λ* < 0, it is an attenuation term without oscillation; when *λ* is a real number and *λ* > 0, it is a rising term without oscillation. Generally, the resonant frequency of the gyroscope is locked by the phase-locked loop, ensuring that the frequency detuning is limited within a small range. As a result, *λ* can be guaranteed to be a real number. According to Equation (34), the simulation results of the gyroscopes responses are displayed in Figure 5.

Obviously, when *λ* < 0, the time-varying term will decrease quickly, and the final expression of the response is an approximate double frequency signal; while when *λ* > 0, the response is a mixing wave of the fundamental frequency and the double frequency signal, whose amplitude will show an exponential increase with time as shown in Figure 5.

## 5. Experimental Results

**Figure 6:**
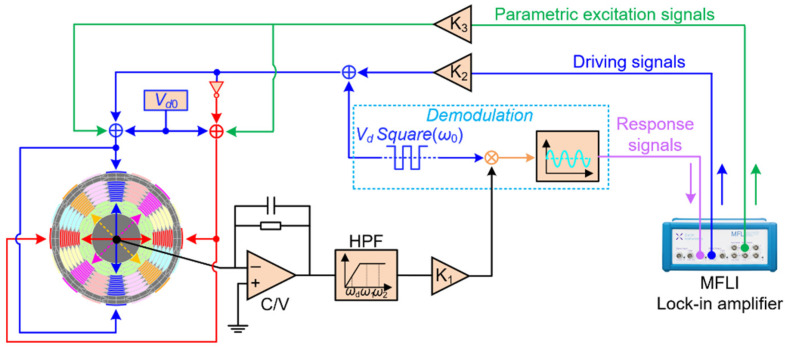
Schematic diagram of the closed-loop non-differential parametric amplification.

To carry out parametric excitation experiments, a lock-in amplifier is used to generate the excitation signals and tune the phase difference between the driving signal and the parametric pump. The diagram of the closed-loop non-differential parametric excitation experiment settings is demonstrated in Figure 6. The diagram for triple frequency parametric excitation is similar to Figure 6, except that its pump signals and driving signals pass through the inverter at the same time.

The key parameters of the DRG are shown in Table 1.

### 5.1. The Reduction of the Driving Voltage

**Figure 7:**
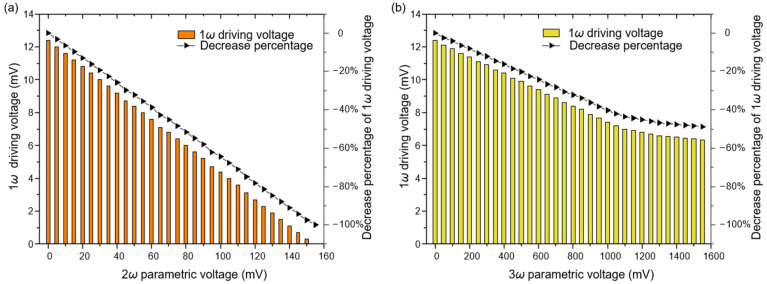
The driving voltage *V_d_* and its decrease percentage varying with different parametric excitation voltage *V_p_*. The oscillation amplitude was maintained at 500 mV by a PID controller (the displacement of the ring is approximately 3 μm). (**a**) Non-differential parametric excitation method; (**b**) triple frequency parametric excitation method.

In order to verify the methods proposed in Section 3.2, we keep the gyroscope vibrating at a certain amplitude and compare the required magnitude of the driving voltage *V_d_* under different parametric excitation methods. For the same vibration amplitude and parametric voltage *V_p_*, the higher the efficiency of parameter amplification, the smaller the driving voltage *V_d_* should be required to be. The experimental results shown in Figure 7 demonstrate that both the triple-frequency method and the non-differential method are effective for parametric amplification, and the non-differential method reduces the 1*ω* driving voltage by a larger magnitude for the same value of the parametric voltage. For example, eighty millivolts are required for a 50% reduction of driving voltage in Figure 7a while more than 1600 mV are required in Figure 7b, which means the higher potential for the non-differential parametric excitation method.

### 5.2. The Stability Boundary and Threshold Voltage

**Figure 8:**
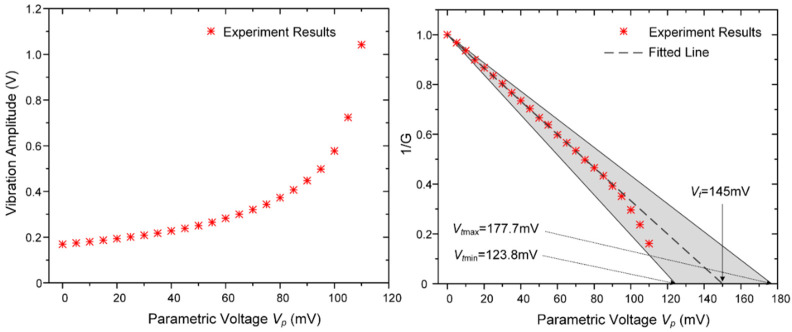
The gyroscope’s response and the gain factor at different parametric pump voltages (non-differential method). The 1*ω* driving voltage is maintained at 10 mV, and the phase of *V_p_*(*t*) is ahead of *V_d_*(*t*) by *π*. The minimum and maximum theoretical value of the threshold voltage are determined by the errors of the DRG’s parameters shown in Table 1.

In order to evaluate the parametric gain and to find the stability boundary experimentally, different parametric pump voltages are applied on the driven electrodes while keeping the fundamental frequency driving voltage at a constant level (10 mV). The gyroscope’s response amplitudes at different pump voltages are recorded and compared with the theoretical results as shown in Figure 8.

It is apparent that the vibration amplitude increases with the increment of the parametric voltage *V_p_*, and the reciprocal of the parametric gain factor 1/*G* has an approximately linear relationship with the parametric voltage *V_p_*, which is consistent with the theory. However, the parametric gain increases sharply especially when the parametric voltage exceeds 90 mV. This is because the terms of higher orders (*j* ≥ 2) in Equation (4) play an important role in the electrostatic driving force when the gyroscope is working on large displacement, which causes the increment of the electrostatic driving force. Under these circumstances, the steady-state vibration of the gyroscope is broken, and there is no longer a linear relationship between the reciprocal of *G*_1_ and parametric excitation voltage *V_p_*; thus, the parametric gain factor will not satisfy Equation (28).

Furthermore, it can be obtained from Figure 8 that the threshold voltage is about 145 mV by linear extrapolation. When the amplitude of the pump voltage is equal to or exceeds this threshold, the gyroscope will enter a parametric resonance state, and the traditional steady-state amplification condition will be broken.

### 5.3. The Improvement of the “Effective Q”

**Figure 9:**
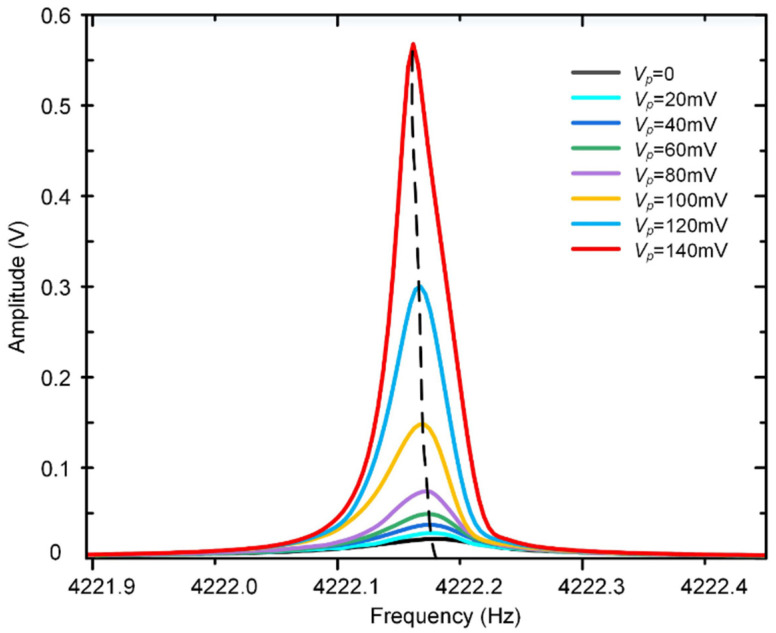
Frequency response for various values of *V_p_*.

In order to display a more intuitive picture of parametric amplification in the non-differential excitation method, a frequency sweep was carried out, as shown in Figure 9. The excitation signal can be expressed by *V*(*t*) = *V_dc_* + *V_d_*sin*ω_d_t* + *V_p_*sin2*ω_d_t*, where *V_dc_* = 6 V and *V_d_* = 2.5 mV. As we can see from Figure 9, the oscillation amplitude is ever-increasing with the increment of *V_p_*. The amplitude reaches 573 mV when *V_p_* = 140 mV, while it is only 24.5 mV when not parametrically amplified, which improved by 23.4 times.

**Figure 10:**
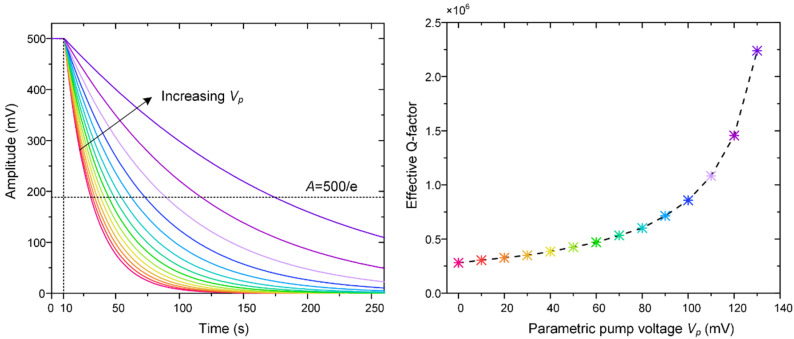
The gyroscope’s attenuation and effective Q-factor with different parametric pump voltages.

However, it is difficult to measure the *Q* factor directly from the frequency response, and the ring-down technique is commonly used in our work for *Q* factor measurement [1]. As a result, to experimentally evaluate the effect of parametric amplification on the “effective *Q*”, the gyroscope’s attenuation curves at the different parametric pump voltages were recorded, as shown in Figure 10. During these experiments, the gyroscope’s oscillation amplitude was maintained at 500 mV, and then, the 1*ω* driving signal was remove, but the parametric pump signal retained. The gyroscope’s oscillation attenuation with different parametric excitation voltages from 0 to 140 mV is shown in Figure 10.

It can be seen from Figure 10 that the “effective *Q*” increases with the increment of the parametric voltage, and the equivalent decaying time is lengthened at the same time. It is obvious that the mechanical sensitivity of the DRG is proportional to its intrinsic quality factor, and it is an inherent attribute of the DRG that cannot be changed by the external signals. However, the output of the DRG will increase with the improvement of “effective *Q*”, which means the improvement of the total sensitivity. Moreover, it should be noted that parametric excitation must be applied in the sense mode for the improvement of sensitivity.

Besides, we found that the gyroscope will keep vibrating all the time even though the 1*ω* driving signal was removed as long as the parametric voltage reaches 145 mV in the experiment, where the DRG enters the parametric resonance condition. In this case, the gyroscope is vibrating under the sole operation of a double resonant frequency signal without the driving energy of the fundamental frequency signal. As a result, this special parametric excitation condition can be used to improve the gyroscope’s response, which reveals a great potential to enhance the sensing capabilities and eliminate crosstalk signals [33].

### 5.4. The Subharmonic Resonance

In the case of subharmonic excitation, the time-varying term is affected by the amplitude of the excitation force as shown in Equation (36). As a result, different changes in the gyroscope’s response under subharmonic excitation can be explored by changing the amplitude of the excitation force. In these series of experiments, the subharmonic AC voltage was set as 0.3 V and 0.7 V, corresponding to the cases of *λ* < 0 and *λ* > 0, respectively.

**Figure 11:**
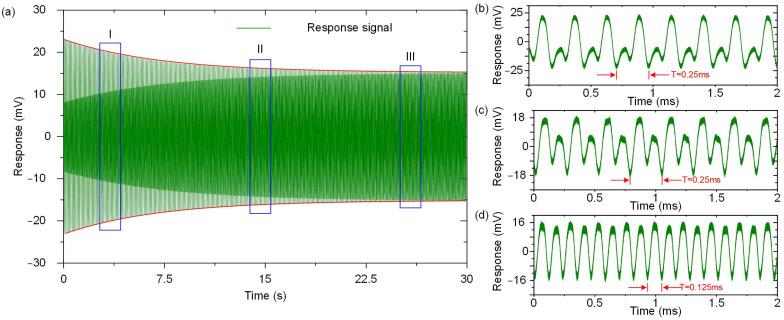
Responses of the disk resonator gyroscope under subharmonic excitation when *λ* < 0. (**a**) The gyroscope’s response in the time domain when *λ* < 0; (**b**) The gyroscope’s response in the I stage; (**c**) The gyroscope’s response in the II stage; (**d**) The gyroscope’s response in the III stage.

In the first case, the amplitude of the AC excitation signal is lower than the threshold, resulting in the amplitude of the fundamental frequency response being an attenuation term without oscillation, as shown in Figure 11a. In the initial stage of the gyroscope’s response, the amplitude of the fundamental frequency response is strong, so the response presents a typically mixed signal of the fundamental frequency and the double frequency as shown in Figure 11b. With the passage of time, the fundamental frequency response gradually becomes weaker as in Figure 11c, and the proportion of the double frequency signal in the mixed-signal increases until it finally shows an approximate double frequency response, as in Figure 11d.

**Figure 12:**
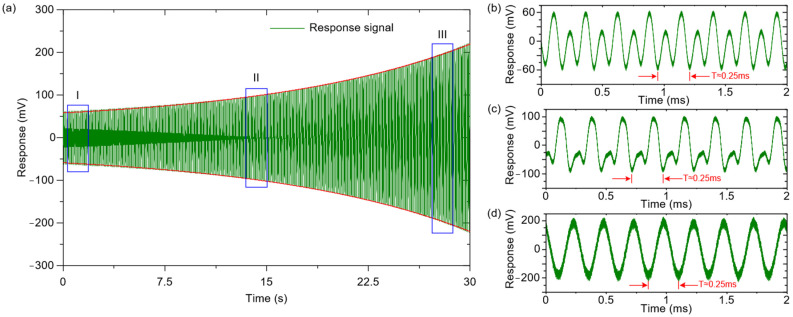
Responses of the disk MEMS gyroscope under subharmonic excitation when *λ* > 0. (**a**) The gyroscope’s response in the time domain when *λ* > 0; (**b**) The gyroscope’s response in the I stage; (**c**) The gyroscope’s response in the II stage; (**d**) The gyroscope’s response in the III stage.

When it comes to the second case, the amplitude of the AC excitation signal is higher than the threshold, resulting in the amplitude of the fundamental frequency response being a rising term without oscillation, as shown in Figure 12a. Compared with the first case, the gyroscope’s response displays an opposite rising trend. In the initial stage of the gyroscope’s response, the fundamental frequency response and the double frequency response are at a comparable level, as shown in Figure 12b. With the passage of time, the fundamental frequency response gradually becomes stronger as in Figure 12c, and its proportion in the mixed-signal increases until it finally shows an approximate fundamental frequency response in Figure 12d.

Obviously, due to the existence of the quadratic nonlinearity, when subharmonic (double frequency) excitation signals are applied on electrodes, the gyroscope’s response converts to a mixed signal of the fundamental frequency and double frequency. Compared with the primary excitation, the subharmonic response proves to have an additional time-varying response in addition to a steady-state response, indicating that it is a combination of different signals and depends on the oscillation time. The evolution of the periodic solution is closely related to the strength of subharmonic excitation signal, which also determines the final manifestation of the gyroscope’s response.

## 6. Conclusions

The parametric and subharmonic excitations in a push-pull driven disk gyroscope are analyzed in this paper. Due to the differential characteristics of push-pull driving method, the traditional parametric excitation method is no longer applicable. Therefore, two novel methods are proposed to take full advantage of both push-pull driving and parametric excitation in this paper.

The first method is to use the triple resonant frequency signal as the parametric pump instead of the traditional double one. In this case, despite the differential effect of the push-pull driven method, the term for double frequency dynamic stiffness modulation generated by the parametric pump signal will still exist and amplify the gyroscope’s response. This method provides an approach to achieve parametric amplification that has never been carried out before.

The second method is to apply the non-differential parametric pump signals to the push-pull driving circuit, making a 7.6 times improvement of the “effective *Q*”. In this method, the pump signals applied to the differential electrodes will not be eliminated, leaving an effective term for double frequency stiffness modulation. The efficiency of this method has improved greatly because the effective term is much bigger than the first method. It is worth noting that whether these two methods are used for amplification or suppression is determined by the phase difference between the fundamental frequency signal and the parametric pump signal.

Moreover, the stability boundary of parametric amplification is analyzed in this paper. The steady-state of the gyroscope will be broken when the parametric voltage reaches the threshold, and it will enter the parametric resonance, where subharmonic excitation plays the key role. In this case, the gyroscope will keep vibrating under the sole action of the parametric pump signals even if the fundamental frequency driven signal has been removed. This is particularly helpful for the gyroscopes where the detection signal is disturbed by the crosstalk signal from drive mode to sense mode generated by the parasitic capacitive. Besides, parametric excitation in sense mode amplifies the Coriolis response directly, which is beneficial for the improvement of sensitivity. This occurs before the addition of the noise of the first electronic stage, which contributes to the noise squeezing. However, this approach is far more demanding on the control system, and future work should address carrying out parametric excitation in the full closed-loop system that includes both drive mode and sense mode.

## Figures and Tables

**Table 1:** Parameters of the DRG.

Parameter	Value
Radius of the outermost ring *r*	4 mm ± 1 μm
Height of the ring *h*	150 μm ± 1 μm
Gap between ring and outer electrodes *d*_0_	12 μm ± 0.5 μm
Effective (modal) mass *m*	1.8 mg ± 0.1 mg
Frequency of drive mode *f*_1_	4222.2 Hz
Frequency of sense mode *f*_2_	4220.1 Hz
Quality factor of drive mode *Q*_1_	292,995
Quality factor of sense mode *Q*_2_	286,489

## References

[1] Li Q., Xiao D., Zhou X., Xu Y., Zhuo M., Hou Z., He K., Zhang Y., Wu X. (2018). 0.04 degree-per-hour MEMS disk resonator gyroscope with high-quality factor (510 k) and long decaying time constant (74.9 s). Microsyst. Nanoeng..

[2] Bao M., Yang H. (2007). Squeeze film air damping in MEMS. Sens. Actuators Phys..

[3] Unterreithmeier Q.P., Faust T., Kotthaus J.P. (2010). Damping of nanomechanical resonators. Phys. Rev. Lett..

[4] Zhou X., Xiao D., Li Q., Hu Q., Hou Z., He K., Chen Z., Zhao C., Wu Y., Wu X. (2018). Investigation on the quality factor limit of the (111) silicon based disk resonator. Micromachines.

[5] Turner K.L., Miller S.A., Hartwell P.G., MacDonald N.C., Strogatz S.H., Adams S.G. (1998). Five parametric resonances in a microelectromechanical system. Nature.

[6] Rugar D., Grütter P. (1991). Mechanical parametric amplification and thermomechanical noise squeezing. Phys. Rev. Lett..

[7] Karabalin R.B., Lifshitz R., Cross M.C., Matheny M.H., Masmanidis S.C., Roukes M.L. (2011). Signal amplification by sensitive control of bifurcation topology. Phys. Rev. Lett..

[8] Zhang W., Baskaran R., Turner K.L. (2002). Effect of cubic nonlinearity on auto-parametrically amplified resonant MEMS mass sensor. Sens. Actuators Phys..

[9] Kacem N., Hentz S., Baguet S., Dufour R. (2011). Forced large amplitude periodic vibrations of non-linear mathieu resonators for microgyroscope applications. Int. J. Non-Linear Mech..

[10] Tiwari S., Candler R.N. (2019). Using flexural MEMS to study and exploit nonlinearities: A review. J. Micromech. Microeng..

[11] Sobreviela G., Zhao C., Pandit M., Do C., Du S., Zou X., Seshia A. (2017). Parametric noise reduction in a high-order nonlinear MEMS resonator utilizing its bifurcation points. J. Microelectromech. Syst..

[12] Han M., Zhang Q., Hao S., Li W. (2019). Parametric characteristics and bifurcation analysis of multi-degree-of-freedom micro gyroscope with drive stiffness nonlinearity. Micromachines.

[13] Su Y., Xu P., Han G., Si C., Ning J., Yang F. (2020). The characteristics and locking process of nonlinear MEMS gyroscopes. Micromachines.

[14] Zhou X., Zhao C., Xiao D., Sun J., Sobreviela G., Gerrard D.D., Chen Y., Flader I., Kenny T.W., Wu X. (2019). Dynamic modulation of modal coupling in microelectromechanical gyroscopic ring resonators. Nat. Commun..

[15] Mahboob I., Yamaguchi H. (2008). Piezoelectrically pumped parametric amplification and Q enhancement in an electromechanical oscillator. Appl. Phys. Lett..

[16] Harish K.M., Gallacher B.J., Burdess J.S., Neasham J.A. (2009). Experimental investigation of parametric and externally forced motion in resonant MEMS sensors. J. Micromech. Microeng..

[17] Nayfeh A.H., Mook A.D. (1979). Nonlinear Oscillations.

[18] Ahn C.H., Nitzan S., Ng E.J., Hong V.A., Yang Y., Kimbrell T., Horsley D.A., Kenny T.W. (2014). Encapsulated high frequency (235 kHz), high-Q (100 k) disk resonator gyroscope with electrostatic parametric pump. Appl. Phys. Lett..

[19] Gallacher B.J., Burdess J.S., Harish K.M. (2006). A control scheme for a MEMS electrostatic resonant gyroscope excited using combined parametric excitation and harmonic forcing. J. Micromech. Microeng..

[20] Hu Z.X., Gallacher B.J., Burdess J.S., Fell C.P., Townsend K. (2011). A parametrically amplified MEMS rate gyroscope. Sens. Actuators Phys..

[21] DeMartini B.E., Rhoads J.F., Turner K.L., Shaw S.W., Moehlis J. (2007). Linear and nonlinear tuning of parametrically excited MEMS oscillators. J. Microelectromech. Syst..

[22] Oropeza-Ramos L.A., Burgner C.B., Turner K.L. (2009). Robust micro-rate sensor actuated by parametric resonance. Sens. Actuators Phys..

[23] Sharma M., Sarraf E.H., Baskaran R., Cretu E. (2012). Parametric resonance: Amplification and damping in MEMS gyroscopes. Sens. Actuators Phys..

[24] Nitzan S.H., Zega V., Li M., Ahn C.H., Corigliano A., Kenny T.W., Horsley D.A. (2015). Self-induced parametric amplification arising from nonlinear elastic coupling in a micromechanical resonating disk gyroscope. Sci. Rep..

[25] Harish K.M., Gallacher B.J., Burdess J.S., Neasham J.A. (2008). Simple parametric resonance in an electrostatically actuated microelectromechanical gyroscope: Theory and experiment. Arch. Proc. Inst. Mech. Eng. Part C J. Mech. Eng. Sci..

[26] Miller J.M.L., Ansari A., Heinz D.B., Chen Y., Flader I.B., Shin D.D., Villanueva L.G., Kenny T.W. (2018). Effective quality factor tuning mechanisms in micromechanical resonators. Appl. Phys. Rev..

[27] Zheng X., Wu H., Lin Y., Ma Z., Jin Z. (2020). Linear parametric amplification/attenuation for MEMS vibratory gyroscopes based on triangular area-varying capacitors. J. Micromech. Microeng..

[28] Awrejcewicz J. (2014). Applied Non-Linear Dynamical Systems.

[29] Xu Y., Li Q., Zhang Y., Zhou X., Wu X., Xiao D. (2020). Honeycomb-like disk resonator gyroscope. IEEE Sens. J..

[30] Acar C., Shkel A. (2009). MEMS Vibratory Gyroscopes; MEMS Reference Shelf.

[31] Szemplińska-Stupnicka W. (1978). The generalized harmonic balance method for determining the combination resonance in the parametric dynamic systems. J. Sound Vib..

[32] Li Q., Sun J., Xu Y., Wang P., Zhou X., Lu K., Wu X., Xiao D. A novel nonlinearity reduction method in disk resonator gyroscopes based on thevibration amplification effect. Proceedings of the 20th International Conference on Solid-State Sensors.

[33] Dolleman R.J., Houri S., Chandrashekar A., Alijani F., van der Zant H.S.J., Steeneken P.G. (2018). Opto-thermally excited multimode parametric resonance in graphene membranes. Sci. Rep..

